# Precision Oxygen Therapy in the Intensive Care Unit: Matching Oxygen Exposure to Patient Phenotypes

**DOI:** 10.3390/jpm16030158

**Published:** 2026-03-12

**Authors:** Jhon Jairo Botello Jaimes, Angie Katherine Turriago Castañeda, Kevin Fernando Montoya-Quintero, Johana Galván Barrios

**Affiliations:** 1 Facultad de Ciencias de la Salud, Corporación Universitaria Remington, Medellin 050012, Colombia; jhon.botello@uniremington.edu.co; 2 Universidad ICESI, Cali 760031, Colombia; angieturriago.65@gmail.com; 3 Departamento Médico, AT Salud Integral SAS, Medellin 050001, Colombia; 4 Facultad de Ciencias para la Salud, Universidad de Manizales, Manizales 170001, Colombia; kmontoya@umanizales.edu.co; 5 Biomedical Scientometrics and Evidence-Based Research Unit, Department of Health Sciences, Universidad de la Costa, Barranquilla 080002, Colombia

**Keywords:** oxygen inhalation therapy, intensive care units, critical care, meta-research, evidence gaps, evidence-based practice

## Abstract

Oxygen therapy is one of the most widely used interventions in critical care, yet it remains poorly individualized. Recent trials and meta-analysis suggest no mortality difference between conservative and liberal oxygen strategies, reinforcing the perception that dose does not matter within usual ranges. From this perspective, we argue that this apparent neutrality may largely reflect methodological and conceptual limitations, although true clinical equivalence in some patient populations remains plausible and cannot be excluded based on current evidence. Heterogeneous populations, overlapping oxygenation targets, and the absence of exposure metrics (time in hyperoxia, time in hypoxemia, and cumulative partial pressure of arterial oxygen/peripheral oxygen saturation curves) dilute phenotype-specific signals and force distinct physiological responses into a single pooled estimate. We propose a conceptual model in which oxygen behaves as a dose-dependent, time-dependent drug with phenotype-specific therapeutic windows, particularly in chronic hypercapnia, traumatic brain injury, sepsis, and early versus late acute respiratory distress syndrome. Building on this model, we outline a methodological agenda for precision oxygen trials: defining interventions by actual exposure, pre-specifying pathophysiological subgroups, adopting patient-centered core outcome sets, and using adaptive, target-range designs and individual patient data meta-analyses. For contemporary guidelines and research, the key question is no longer whether conservative or liberal oxygen therapy is superior on average, but how to match the right oxygenation range to the right intensive care unit phenotype at the right time. Moving from population-averaged comparisons to exposure-aware, phenotype-oriented strategies is essential if oxygen therapy is to become a truly precision intervention in critical care.

## 1. The Problem of Average Effects in Oxygen Therapy Research

Oxygen therapy is one of the most frequently used interventions in the intensive care unit (ICU), and yet paradoxically one of the least individualized. Although the physiological consequences of both hypoxemia and hyperoxia are well described [1], most clinical trials still conceptualize oxygen therapy as a binary choice, conservative versus liberal, as if all critically ill patients shared a similar tolerance to oxygen exposure [2]. This simplification has shaped the design of randomized controlled trials (RCTs) and subsequent meta-analyses [3], many of which report neutral or minimal differences in mortality when comparing broad oxygenation strategies across diverse ICU populations [3].

The most recent synthesis of these data, the 2024 meta-analysis by Li and colleagues [3], offers a rigorous and valuable assessment of existing trials. Its primary conclusion, that conservative and liberal oxygen therapy strategies do not differ significantly in overall mortality, has been interpreted by some as evidence that oxygen dosing may matter less than once assumed [3].

For the average patient enrolled in these studies, this interpretation may well be accurate. Yet clinicians at the bedside know that critically ill patients are anything but average [4]. Their physiology, comorbidities, and susceptibilities to oxygen-related harm vary widely, and such differences can meaningfully influence the risk-benefit balance of any given oxygenation strategy [5].

This tension reflects not a flaw in any single study, but rather a broader epistemological challenge: evidence synthesis frameworks are built to estimate population-level effects, not to capture the nuanced, phenotype-specific responses seen in real-world critical care [6]. Meta-analyses strive to generate a summary effect under the assumption that included studies address a common clinical question [7].

However, oxygen therapy trials differ substantially in the populations they enroll (from sepsis and early Acute Respiratory Distress Syndrome [ARDS] to neurocritical care and postoperative patients) [8], the oxygenation targets they employ (Peripheral Oxygen Saturation [SpO_2_] thresholds, Partial Pressure of Arterial Oxygen [PaO_2_] goals, or Fraction of Inspired Oxygen [FiO_2_] titration), and the duration and intensity of exposure to hyperoxia or hypoxemia [9]. When such heterogeneity is aggregated, the resulting pooled estimate becomes a statistical average that may not represent any specific patient subgroup.

This raises an essential question: Does a neutral average effect truly reflect clinical equipoise, or does it obscure opposing effects among physiologically distinct subpopulations? From a physiological standpoint, divergent treatment effects are not only plausible, but expected. Patients with chronic hypercapnia, traumatic brain injury, septic microvascular dysfunction, or early versus late ARDS likely have different thresholds for both oxygen toxicity and oxygen debt [10]. Yet these phenotypes are rarely analyzed in depth within trial-level reports and become effectively invisible when studies are combined using traditional meta-analytic techniques.

A second limitation arises from the way oxygen therapy is operationalized. Labels such as conservative and liberal convey a sense of contrast, but the underlying exposure often overlaps substantially between study arms [3]. A conservative target may still allow prolonged periods of SpO_2_ > 98%, whereas a liberal strategy may include extensive time within normoxic ranges.

Without capturing more granular exposure metrics, time in hyperoxia, time in hypoxemia, variability in SpO_2_ or PaO_2_, and cumulative dose (e.g., area under the curve of oxygen tension), we cannot determine whether trial participants truly experienced distinct interventions [11]. Consequently, meta-analyses may therefore conclude that oxygen strategy does not influence outcomes when, in some cases, the interventions under comparison may not have been physiologically different enough to meaningfully alter risk [12].

Thus, the challenge is not that global meta-analyses are incorrect, but that they are inherently insufficient to inform precision decision-making in the ICU. Oxygen behaves less like a simple supportive therapy and more like a pharmacologic exposure with a therapeutic window, toxicity thresholds, and time-dependent effects [13]. Traditional evidence synthesis methods are not always designed to fully capture these dimensions, particularly when applied to heterogeneous patient populations with widely differing physiological phenotypes [14].

As precision medicine reshapes clinical practice across multiple specialties, the expectation that a single oxygenation strategy can be applied uniformly to all critically ill patients appears increasingly misaligned with biological reality. What matters to patients is not the average effect of an intervention across thousands of individuals, it is whether the specific dose and exposure pattern they receive is appropriate for their physiology.

In this perspective, we argue that the next phase of oxygen therapy research must move beyond broad population averages and embrace a phenotype-specific, exposure-aware model of precision oxygenation. We outline a conceptual framework that treats oxygen as a dose-dependent therapy, identify methodological priorities for future trials and meta-analyses, and explore the implications for adaptive clinical designs and evidence-based guidelines. Our central premise is that advancing oxygen therapy requires aligning research methods with the biological diversity of the ICU population.

This perspective does not aim to systematically review the literature, but rather to critically reinterpret contemporary high-quality evidence, particularly recent RCTs and meta-analyses, through a physiological and meta-research framework relevant to precision medicine in critical care.

## 2. Why the 2024 Meta-Analysis Matters: Yet Why It Cannot Close the Debate

In this section, we briefly summarize and critically contextualize contemporary high-quality evidence as a foundation for the subsequent conceptual arguments.

The meta-analysis by Li et al. [3] represents the most comprehensive and up-to-date synthesis of randomized evidence comparing conservative oxygen therapy with liberal oxygen therapy in ICU patients. By incorporating 13 RCTs and 10,632 participants, this review provides an unusually large information base for a field historically dominated by small, heterogeneous studies. Its central quantitative finding is clear: no significant difference in mortality between conservative oxygen therapy and liberal oxygen therapy at any standard time point [3].

Specifically, the pooled risk ratios were almost perfectly neutral: (a) 30-day mortality: Relative Risk [RR] 1.01 (95% CI: 0.94–1.09; I^2^ = 42%); (b) 90-day mortality: RR 1.01 (95% CI: 0.95–1.08; I^2^ = 9%); and (c) Longest follow-up mortality: RR 1.00 (95% CI: 0.95–1.06; I^2^ = 22%).

These effect estimates, with confidence intervals tightly centered around unity, strongly suggest that, at the population level, neither strategy conveys a meaningful survival advantage. The accompanying trial sequential analysis further reinforces this interpretation: with 85.89% of the required information size accrued (9614 of 11,194 patients), the cumulative Z-curve crossed the futility boundary, supporting the conclusion that a 20% relative mortality reduction with conservative oxygen therapy is unlikely [3].

This is an important and methodologically robust outcome. It provides clinicians and guideline developers with reassurance that, across broad ICU populations, targeting modestly lower versus higher oxygenation levels does not produce large survival differences [3]. In an era where overinterpretation of small trials has previously fueled controversy, this updated synthesis gives the field a much-needed stabilizing evidence anchor.

However, the very strength of this meta-analysis, its breadth and aggregation, also exposes its key limitation. Neutrality at the aggregate level does not necessarily imply neutrality within clinically relevant subgroups. This point emerges repeatedly upon closer inspection of the underlying data.

### 2.1. Heterogeneous Populations Dilute Phenotype-Specific Signals

The meta-analysis intentionally pooled trials enrolling diverse populations: mixed ICU cohorts, medical ICU cohorts, mild-to-moderate hypoxemia, moderate-to-severe hypoxemia, septic shock, early ARDS, Coronavirus Diseases 2019 (COVID-19) respiratory failure, and postoperative or undifferentiated critical illness [3]. Yet oxygen physiology is not uniform across these conditions [13].

The subgroup analyses reported in the review found no statistically significant differences, but the point estimates varied substantially. For example: In several medical ICU studies enrolling predominantly respiratory failure patients, point estimates suggested higher mortality in the conservative oxygen therapy group, particularly when PaO_2_ < 80 mmHg was achieved [3].

Conversely, in mixed ICU settings, including influential trials, conservative oxygen therapy showed numerically lower mortality, although confidence intervals crossed 1 [3].

These opposing tendencies may partially offset each other in pooled estimates, producing a summary result that hides meaningful physiological heterogeneity. The absence of statistical significance should not be conflated with the absence of clinically relevant effect modification.

### 2.2. Conservative and Liberal Strategies Overlap Substantially Across Trials

The exposure contrast between conservative oxygen therapy and liberal oxygen therapy was, in many cases, far narrower than their labels suggest (PaO_2_ in the conservative oxygen therapy arms ranged from 61 to 87 mmHg, while PaO_2_ in the liberal oxygen therapy arms ranged from 76 to 115 mmHg) [3].

This means that in several trials, the upper range of conservative oxygen therapy overlapped with the lower range of liberal oxygen therapy, and in others the conservative arm still spent substantial time above PaO_2_ 80 mmHg, well within what many would consider normoxic or mildly hyperoxic ranges [3].

Thus, the intervention contrast may not have been sufficiently distinct to test the underlying physiological hypothesis: that hyperoxia harms certain phenotypes, and that preventing it improves outcomes.

### 2.3. Time in Target and Cumulative Oxygen Exposure Were Not Measured

Perhaps a key methodological limitation, and one repeatedly highlighted in oxygen therapy research, is the absence of metrics such as: (a) Time in hyperoxia; (b) Time in hypoxemia; (c) Time in the intended SpO_2_/PaO_2_ range; and (d) Area under the curve (AUC) for PaO_2_ [15].

Without these exposure metrics, the meta-analysis cannot ascertain whether the intended physiological separation between conservative oxygen therapy and liberal oxygen therapy was achieved [15]. Two patients with identical mean PaO_2_ may have profoundly different exposure profiles: one experiencing brief hyperoxic spikes, the other prolonged moderate hyperoxia, yet these are treated as equivalent in meta-analytic pooling.

### 2.4. Adverse Events Suggest Potential Physiological Asymmetry

Interestingly, the meta-analysis reported a lower incidence of adverse events in the conservative oxygen therapy group. Although these findings do not translate into a mortality benefit, they may reflect a potential physiological asymmetry in oxygen-related harm [16]. However, these observations must be interpreted with caution. Definitions of adverse events varied across trials, and the analyses were not designed to establish causal relationships [16]. Consequently, these findings should be viewed primarily as hypothesis-generating signals, consistent with known pathophysiological mechanisms, rather than as definitive evidence of benefit or harm [3].

### 2.5. Why the Debate Remains Open

The meta-analysis by Li et al. [3] is a cornerstone in the field, but it does not close the debate on optimal oxygenation in ICU care, because it addresses a question that may be insufficiently granular for a highly heterogeneous population. It tells us that:

- Across mixed ICU populations, broadly defined conservative oxygen therapy and liberal oxygen therapy strategies do not differ in average mortality.
- But it cannot tell us which phenotypes benefit from lower oxygenation,
- Nor which are harmed by it,
- Nor how actual exposure (not targets) shapes outcomes,
- Nor whether precision oxygen therapy, personalized by physiological phenotype, outperforms categorical strategies [3].

**Figure 1:**
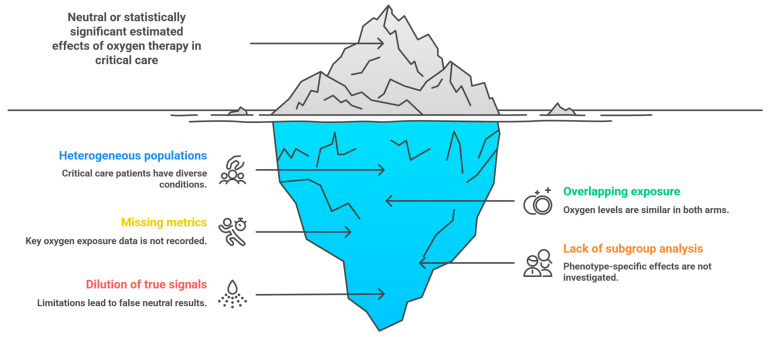
Conceptual and methodological limitations underlying the neutral/significant effects findings of current oxygen therapy trials and meta-analyses. Clinical trials comparing conservative versus liberal oxygen strategies frequently enroll heterogeneous ICU populations, apply overlapping oxygenation targets, and lack standardized exposure metrics such as time in hyperoxia, time in hypoxemia, and cumulative PaO_2_/SpO_2_ dose. These limitations dilute phenotype-specific signals and produce pooled estimates that appear neutral, even when clinically meaningful benefits or harms may exist within distinct physiological subgroups. Collectively, these methodological constraints explain why contemporary meta-analyses show no overall mortality difference and highlight the need for exposure-based and phenotype-oriented research designs. Source: authors.

In essence, the meta-analysis answers a population-level question in a field where individual physiology plays a critical role in shaping risk, even when average effects appear neutral [13]. This is not a limitation of the authors’ methods; it is a limitation of the evidence ecosystem as currently constructed (Figure 1).

Importantly, the arguments presented above operate at different levels of evidence. Physiological reasoning provides hypothesis-generating insights into why divergent responses to oxygen exposure are biologically plausible. Empirical subgroup trends observed in RCTs and meta-analyses offer suggestive, but non-causal, signals that such heterogeneity may exist. By contrast, established causal inference remains grounded in population-level randomized evidence, which to date has largely demonstrated neutral average effects. Recognizing these distinctions is essential to avoid overinterpretation while still acknowledging the limitations of aggregated analyses (Table 1).

## 3. A Conceptual Model: Oxygen as a Dose-Dependent Exposure with Phenotype-Specific Thresholds

Building on the empirical limitations outlined above, this section advances a conceptual model that reframes oxygen therapy as a dose-dependent, time-varying, and phenotype-specific intervention. The physiological mechanisms underlying oxygen-related benefit and harm are outlined here as the central conceptual foundation of the manuscript and are referenced throughout subsequent sections.

Despite its routine use in critical care, oxygen is not a neutral background therapy, it is a biologically active drug whose effects follow well-defined dose–response relationships [13]. Both insufficient and excessive oxygen delivery can trigger injury pathways, but the shape of this dose–response curve varies profoundly across clinical phenotypes [15,16]. Recognizing oxygen as a time-dependent exposure with phenotype-specific toxicity thresholds is essential to understanding why population-level trials yield neutral results while individual patients exhibit dramatically different responses [17].

### 3.1. Oxygen Has a Therapeutic Window, Not a Binary “More vs. Less” Effect

Traditional RCTs implicitly assume that oxygen is either helpful or harmful in a linear fashion [10,14]. Yet the underlying physiology suggests a U-shaped relationship:

- Hypoxemia (low PaO_2_/SpO_2_) produces oxygen debt, impaired oxidative phosphorylation, acidosis, organ dysfunction, and eventually cellular death [13,18,19].
- Hyperoxia (supraphysiologic PaO_2_) promotes reactive oxygen species, endothelial dysfunction, mitochondrial injury, vasoconstriction (especially cerebral), immune dysregulation, and ventilator-induced lung injury amplification [13,18,19].

Thus, optimal oxygenation lies within a narrow therapeutic window, which may shift depending on comorbidities, acute pathophysiology, and even disease stage [13,18,19].

This concept directly contrasts with the conservative vs. liberal dichotomy used in most trials, where exposure distributions often overlap and fail to reflect the true biological gradient of toxicity or benefit.

### 3.2. Oxygen Exposure Is Cumulative and Time-Dependent

The traditional approach of reporting a single target SpO_2_ range or mean PaO_2_ does not capture the dynamic nature of oxygen delivery [14]. What matters biologically is not an average value, but the cumulative exposure over time: (a) Time spent in hyperoxia; (b) Time spent in hypoxemia; (c) Variability/fluctuation amplitude; and (d) Cumulative AUC of PaO_2_ or SpO_2_ [13,19].

For example, a patient with intermittent hyperoxic spikes (PaO_2_ 150–200 mmHg) may have a vastly different risk profile than a patient who stays steadily at PaO_2_ 90–100 mmHg, even if their mean oxygenation level appears similar.

Current RCT designs rarely capture these trajectories, and meta-analyses cannot incorporate them, leading to the misleading impression that oxygen exposure is simple and interchangeable across patients.

### 3.3. Different Phenotypes Have Different Thresholds for Oxygen Toxicity and Oxygen Debt

One of the central reason’s population-level trials yield neutral effects is that oxygen behaves differently depending on the underlying pathophysiology [20]. Below are the major phenotypes with distinct response curves:

- Chronic hypercapnia/Chronic Obstructive Pulmonary Disease (COPD): Patients with chronic CO_2_ retention show heightened susceptibility to oxygen-induced hypercapnia (via ventilation–perfusion mismatch and hypoventilation), narrowing the safe therapeutic window [21,22]. Even modest PaO_2_ elevations may precipitate clinically relevant deterioration [21,22].
- Traumatic brain injury and acute neurological injury: Hyperoxia can induce cerebral vasoconstriction and reduce cerebral blood flow, raising concern for oxygen-related harm in vulnerable neurovascular states [23]. Avoiding sustained PaO_2_ >120–150 mmHg may therefore be particularly relevant in this subgroup [23].

Sepsis and septic shock: Sepsis disrupts microvascular perfusion and mitochondrial function, such that excess oxygen may amplify oxidative stress without reliably improving tissue oxygen delivery [24]. Nonetheless, in profound hypoperfusion, short, carefully monitored higher oxygen exposure may have context-dependent value, an effect easily obscured in aggregate analyses [24].

- ARDS across stages (early vs. late): Early ARDS is often shunt-dominant and may require higher FiO_2_ to maintain adequate oxygenation, whereas later ARDS may tolerate lower targets as oxygen toxicity becomes a more prominent risk [25]. Pooling these stages within the same syndrome can mask opposing physiological needs [25].
- COVID-19 and viral pneumonitis phenotypes: Early in the COVID-19 pandemic, distinct respiratory phenotypes were described, including Type L (low elastance, preserved compliance) and Type H (high elastance, ARDS-like physiology), each with different oxygenation and perfusion characteristics. Distinct compliance and perfusion patterns imply different oxygen requirements and potentially different susceptibility to hyperoxic lung injury [26,27]. This heterogeneity may help explain inconsistent trends across COVID-19 oxygen studies [26,27,28].

### 3.4. The Idea of a Universal Oxygen Target Appears Increasingly Difficult to Reconcile with Current Physiological Understanding

Given these contrasting dose–response curves: A PaO_2_ of 75 mmHg may be safe and desirable for most patients, but insufficient for early ARDS with severe shunt, and excessive for COPD or traumatic brain injury [21,23,25,27].

Similarly, a PaO_2_ of 100–120 mmHg, widely seen in liberal arms of trials, may be harmless for postoperative surgical patients but dangerous for those with sepsis, traumatic brain injury, or chronic lung disease [10,14].

Under these conditions, average effects are likely to appear neutral when interventions benefit some phenotypes while harming others, even though true equivalence across certain populations may exist. This represents a major limitation of current meta-analytic practice: heterogeneous dose–response relationships cannot be meaningfully summarized into a single point estimate.

### 3.5. The Conceptual Shift: Oxygen as a Precision Therapy

Taken together, these observations support a new conceptual paradigm: Oxygen therapy must be understood as a dose-dependent, phenotype-specific exposure that requires individualized titration, not a categorical strategy applied uniformly.

This reframing provides a coherent explanation for (a) the neutral results of the 2024 meta-analysis [3]; (b) the inconsistent magnitude and direction of individual trials; and (c) the persistently unresolved clinical uncertainty.

It also creates the intellectual foundation for the next section: a methodological agenda that aligns clinical trial design with the underlying biology rather than arbitrary saturation categories. This conceptual framework provides the physiological rationale upon which the subsequent methodological and translational considerations are built, avoiding repeated mechanistic exposition while preserving biological coherence.

## 4. Methodological Agenda: Towards Trials and Meta-Analyses with Better Knowledge Translation

The purpose of the following methodological agenda is not to elevate physiological plausibility or subgroup signals to causal evidence, but to outline research strategies capable of formally testing these hypotheses within appropriate experimental or quasi-experimental designs.

The neutral findings of the meta-analysis by Li et al. [3] underscore a fundamental problem: the current methodological approaches are misaligned with the physiological complexity of oxygen therapy [13,29,30]. If we continue to design trials around broad categorical targets and perform meta-analyses that pool incompatible phenotypes and exposures, we may continue to generate answers that are statistically sound yet only partially informative for clinical decision-making.

A genuine advance in the field requires rethinking not only what we study, but how we measure, analyze, and synthesize oxygen therapy. Below, we outline a methodological agenda designed to produce evidence that can be directly translated into practice and guide precision oxygen strategies.

### 4.1. Define Oxygenation Interventions Based on Actual Exposure, Not Nominal Targets

Most RCTs define conservative oxygen therapy and liberal oxygen therapy by intended SpO_2_ or PaO_2_ ranges [10,14], yet the actual exposure delivered frequently overlaps between arms. This makes it nearly impossible to detect true biological signals.

Future trials must report at least four exposure metrics:

- Time in target range;
- Time spent in hyperoxia (e.g., SpO_2_ > 96% or PaO_2_ > 100–120 mmHg based on phenotype);
- Time spent in hypoxemia (e.g., SpO_2_ < 88–92% depending on condition);
- Cumulative oxygen exposure (AUC-PaO_2_ or AUC-SpO_2_).

These metrics would allow clinicians to understand the relationship between true oxygen exposure and outcomes, something that current evidence cannot provide.

Without quantifying exposure, oxygen therapy trials risk comparing interventions that are biologically indistinguishable despite different labels [13].

### 4.2. Pre-Specify Phenotype-Based Subgroups Grounded in Pathophysiology

Rather than treating critically ill patients as a single homogeneous entity, trials must stratify randomization and analysis by physiologically meaningful categories:

- Chronic hypercapnia/COPD;
- Traumatic brain injury and neurological injury;
- Sepsis and septic shock;
- Early vs. late ARDS;
- COVID-19 viral pneumonitis phenotypes;
- Postoperative vs. medical ICU patients.

These groups exhibit different thresholds for oxygen toxicity and oxygen debt [21,22,23,24,25,26,27].

Pooling them without pre-specified stratification, something common in past trials and meta-analyses, is likely to dilute clinically relevant effects. Moreover, post hoc subgroup analyses are insufficient; they must be embedded in the statistical plan with adequate power.

It should be acknowledged that clinical phenotypes often overlap, evolve over time, and may be difficult to identify prospectively, which represents a practical challenge for precision-based trial designs.

### 4.3. Adopt Core Outcome Sets That Reflect Patient-Centered and Long-Term Effects

Most RCTs focus heavily on short-term mortality [10,14]. But oxygen exposure affects the following outcomes: neurologic, cognitive, functional, and immunologic [31].

To meaningfully translate evidence into clinical practice, future trials should incorporate standardized core outcomes, including:

- Delirium incidence and duration;
- Neurocognitive function at follow-up;
- Quality of life metrics;
- Days alive and free of ventilation;
- Organ dysfunction trajectories;
- Clinically actionable harms (infection, weakness, arrhythmia) [31].

Hyperoxia- and hypoxemia-induced injuries are not always lethal but may produce morbidity with substantial long-term consequences [31]. Capturing these outcomes provides a much clearer picture of the real impact of oxygen strategies [31].

### 4.4. Incorporate Dynamic, Time-Updated Analyses Instead of Static Group Comparisons

Oxygen therapy is inherently dynamic, yet most trials treat it as a fixed exposure:

- Early titration phase vs. maintenance phase;
- Spontaneous breathing vs. mechanical ventilation;
- Progression of ARDS, sepsis, or neurological injury.

Future trials should integrate time-dependent covariates and dynamic modeling that reflect:

- Changing FiO_2_ needs;
- Evolving lung mechanics;
- Shifts in shock or perfusion states;
- Responsiveness to treatment.

This approach mirrors the real-time decision-making of ICU clinicians and improves knowledge translation.

### 4.5. Use Adaptive and Target-Range Designs Rather than Static Conservative/Liberal Comparisons

Adaptive trials allow adjustment of target ranges based on: patient response, safety signals, or phenotype-specific thresholds. Examples include: (a) Target-range trials (e.g., maintaining patients within evolving PaO_2_ windows); (b) Response-adaptive randomization based on subphenotype performance; and (c) Adaptive enrichment designs, where certain phenotypes are preferentially enrolled as signal emerges.

Such designs prevent exposure to harmful oxygen levels and align more closely with precision medicine strategies [29,31].

### 4.6. Meta-Analyses Must Evolve from Pooling Averages to Modeling Heterogeneity

Traditional pairwise meta-analyses produce a single pooled effect estimate, which, as shown, may be mathematically correct yet clinically misleading.

To improve knowledge translation, future evidence syntheses should incorporate:

- Individual patient data (IPD) meta-analyses. These allow modeling of: exposure curves, time in hyperoxia/hypoxemia, phenotype-specific responses, interaction terms with ventilation, and disease stage [20]. IPD may be the gold standard for oxygen therapy but remains underutilized [20].
- Hierarchical Bayesian models. These better accommodate: between-study differences in targets and exposure, nonlinear dose–response relationships, and context-specific heterogeneity [32].
- Meta-regression using exposure metrics, not labels. Instead of analyzing conservative oxygen therapy vs. liberal oxygen therapy, meta-regression should examine: mean PaO_2_, AUC-PaO_2_, overlap between groups, and distributional skew [33]. This shifts synthesis from category-based to biology-based evidence.

### 4.7. Standardize Reporting of Oxygen Protocols to Enable Reproducibility

Trials should clearly document: titration algorithms, response times to SpO_2_ deviation, criteria for adjusting FiO_2_, staff training and adherence fidelity. Without these, it is impossible to determine whether failures to demonstrate effect reflect: true biological neutrality, or inconsistent implementation of interventions.

Then, improving methodological rigor is not an academic exercise, it is essential to producing evidence that clinicians can trust at the bedside. Oxygen therapy is too biologically complex, and too central to critical care, to rely on broad categories and population averages [29,30,31].

**Figure 2:**
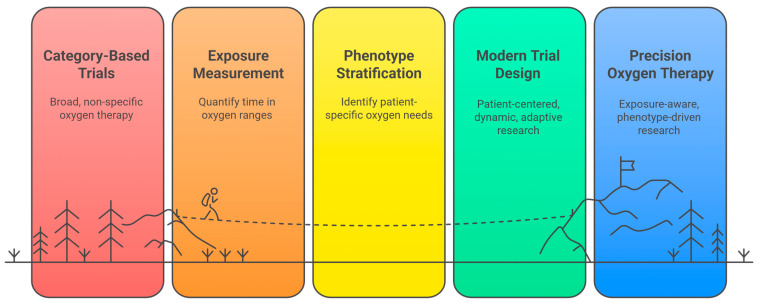
A methodological pathway toward precision oxygen therapy in critical care. The figure depicts the transition from traditional category-based oxygen trials, characterized by broad and overlapping targets, toward a precision framework grounded in biological heterogeneity and actual oxygen exposure. Key methodological components include exposure measurement (time in target and cumulative dose), phenotype stratification across clinically distinct patient groups, and modern trial designs incorporating patient-centered outcomes and time-updated analyses. Together, these elements support an exposure-aware, phenotype-driven approach to oxygen therapy aligned with individual patient physiology. Source: authors.

A methodological shift toward exposure-based, phenotype-aware, dynamic, and patient-centered trial designs is the necessary bridge between physiology and practice (Figure 2).

From a research design perspective, the proposed precision oxygen framework is intentionally conceptual, yet explicitly oriented toward empirical testing. Rather than prescribing a single optimal design, it highlights feasible entry points for future research.

As an initial step, phenotypes characterized by well-established oxygen sensitivity (such as chronic hypercapnia, acute neurological injury, and later-stage ARDS) may represent pragmatic starting points, given their clear pathophysiological rationale and higher signal-to-noise ratio. Within these groups, a minimum feasible exposure set could include time spent above predefined hyperoxic thresholds, time below hypoxemic ranges, and cumulative oxygen exposure metrics (e.g., AUC-PaO_2_ or AUC-SpO_2_), all of which are increasingly available through contemporary ICU monitoring systems.

Regarding study design, multiple complementary approaches appear realistic. While traditional RCTs remain feasible, particularly using target-range or adaptive designs, alternative strategies such as target trial emulation using high-resolution observational data and IPD meta-analyses may offer more immediate opportunities to model exposure-response relationships and phenotype-specific effects. Importantly, these approaches are not mutually exclusive but represent a continuum of methodological options aligned with the complexity of oxygen therapy as a time-varying exposure.

The purpose of this perspective is not to mandate a specific design, but to demonstrate that precision oxygen therapy is not merely an abstract concept; it is a researchable framework that can be progressively operationalized using existing clinical data infrastructures and modern analytical methods.

## 5. Implications for Clinical Guidelines and for Future Target-Range Adaptive Trials

Importantly, the phenotype-aware considerations summarized in Table 2 are aspirational and provisional in nature. They are presented as a conceptual and hypothesis-generating framework rather than as clinical recommendations or guideline directives. Their purpose is to illustrate how physiological heterogeneity might inform future research and clinical reasoning, and any translation into practice will require confirmation in appropriately designed prospective studies (Table 2).

### 5.1. Guidelines Must Move Beyond Categorical SpO_2_/PaO_2_ Targets

Most existing guidelines recommend uniform oxygenation thresholds (e.g., SpO_2_ 92–96%) across diagnoses [34,35]. Given the phenotype-specific differences described earlier, such one-size-fits-all thresholds may not adequately reflect current physiological understanding [21,22,23,24,25,26,27]. Guidelines should begin to integrate:

- Phenotype-aware windows (e.g., narrower upper PaO_2_ limits in traumatic brain injury or COPD; avoidance of deep hypoxemia in early ARDS).
- Explicit acknowledgement of uncertainty where evidence remains heterogeneous.
- Recommendations to minimize unnecessary hyperoxia, guided by the consistent association with adverse events across trials.

Rather than fixed targets, guidelines could adopt a range-based decision framework that emphasizes individual physiology, disease stage, and dynamic oxygen exposure.

### 5.2. Future Trials Should Adopt Target-Range and Adaptive Designs

Static two-arm comparisons are insufficient to capture the complex relationship between oxygen exposure and outcome. Adaptive and range-based designs offer three major advantages:

- Dynamic adjustment to patient response. Trial algorithms can modify oxygen targets in real-time according to: (a) Phenotype (e.g., sepsis vs. neurological injury); (b) Safety signals (e.g., hypoxemia episodes), or (c) Predefined thresholds for hyperoxia. This aligns trial interventions with bedside physiology rather than arbitrary categories.
- Identification of differential treatment effects earlier. Adaptive enrichment allows the trial to preferentially recruit or analyze subgroups demonstrating benefit or harm, helping avoid dilution of meaningful signals.
- Greater separation between exposure curves. Target-range trials explicitly control: (a) Time in target; (b) Time above and below range; and (c) Cumulative exposure metrics. This solves one of the key limitations of past RCTs, where conservative oxygen therapy and liberal oxygen therapy arms frequently overlapped.

### 5.3. Trials and Guidelines Must Align Around Exposure-Based Metrics

Standardizing metrics such as time in hyperoxia, time in hypoxemia, AUC-PaO_2_, and oxygenation variability would allow both researchers and guideline developers to anchor recommendations in actual delivered exposure, not nominal targets. This is the most direct path toward real-world translation of evidence.

### 5.4. Towards a Precision-Oxygenation Paradigm

Ultimately, the goal is not to identify a universally optimal oxygen target, but rather to (1) match the right oxygenation range (2) to the right patient phenotype (3) at the right time in the disease course. Guidelines should reflect this precision framework by encouraging clinicians to titrate oxygen with the same intentionality used for vasoactive drugs or sedation strategies.

From a meta-research perspective, this dilemma exemplifies how methodological simplifications can distort the real causal structure of clinical phenomena [36,37,38,39]. The persistent neutrality observed in population-level meta-analyses is not simply a statistical artifact but a consequence of epistemic misalignment: oxygen therapy is studied as if it were a binary intervention applied to a homogeneous population, rather than a dose-dependent exposure interacting with diverse physiological systems.

This mismatch between the true complexity of the object of study and the reductionist design of clinical trials produces a systematic loss of meaningful signal when heterogeneous realities are forced into overly generalized frameworks [40,41,42,43].

It should also be acknowledged that improvements in overall ICU care, including advances in ventilatory strategies, resuscitation practices, infection control, and nutritional support, may independently influence outcomes commonly attributed to oxygen exposure. These concurrent improvements complicate attribution of outcome differences to oxygen strategy alone and further underscore the need for exposure-aware and context-sensitive research designs.

Population-level neutrality should therefore be interpreted cautiously. In some clinical contexts it may reflect genuine equivalence between strategies, whereas in others it may arise from methodological limitations that dilute phenotype-specific effects. Distinguishing between these possibilities requires confirmatory studies specifically designed to capture exposure-response relationships.

## 6. Conclusions

Current evidence indicates that broadly defined conservative and liberal oxygen strategies do not result in meaningful differences in mortality across the general ICU population. However, this apparent neutrality should not be interpreted as evidence that oxygen therapy is inconsequential, but rather as an indication that current research approaches may not fully capture phenotype-specific effects; it likely reflects, at least in part, the limitations of approaches that average heterogeneous patients, overlapping exposures, and divergent physiological responses.

Oxygen is a biologically active intervention with dose–response effects, relatively narrow therapeutic windows, and phenotype-specific thresholds for harm and benefit. When these complexities are insufficiently captured, trials may fail to detect clinically relevant effects, and meta-analyses may converge toward neutral average estimates. The resulting evidence base can be methodologically robust while remaining incompletely aligned with the nuances of bedside decision-making.

Progress in this field will likely depend on a conceptual shift toward precision-oriented approaches to oxygen therapy, supported by methodological strategies that quantify actual exposure, predefine physiologically informed subgroups, incorporate time-dependent modeling, and emphasize patient-centered outcomes. In this context, future clinical guidelines may benefit from moving beyond universal targets toward more phenotype-aware and exposure-based frameworks.

Rather than further refining the binary debate between conservative and liberal oxygen therapy, a broader reorientation toward aligning research design with underlying biological heterogeneity may be more informative. Such alignment has the potential to generate evidence that is more clinically interpretable and better suited to individualized decision-making in critical care.

Accordingly, any translation of phenotype-aware oxygen strategies into clinical guidelines must await confirmatory evidence from appropriately designed trials and advanced analytical approaches.

## Figures and Tables

**Table 1:** Summary of current high-quality evidence on liberal or higher oxygen strategies in critically ill patients.

Clinical Context/ Population	Oxygen Strategy Evaluated	Potential Merits Reported	Potential Demerits Reported	Key Limitations of the Evidence
Mixed ICU populations	Liberal or higher oxygen targets compared with conservative strategies	Avoidance of severe hypoxemia; reassurance of oxygen reserve in unstable patients	Higher incidence of adverse events in some studies; signals of hyperoxia-related harm	Broad population heterogeneity; overlapping PaO_2_/SpO_2_ ranges between study arms; lack of exposure metrics
Medical ICU patients with respiratory failure	Higher PaO_2_ or SpO_2_ targets	Maintenance of arterial oxygenation during acute respiratory compromise	Trends toward higher mortality in some subgroups; potential oxygen toxicity	Small subgroup sizes; inconsistent phenotype definitions; absence of time-in-range data
Sepsis and septic shock	Liberal oxygen supplementation	Theoretical improvement in oxygen delivery during hypoperfusion	Increased oxidative stress; endothelial and mitochondrial dysfunction	Observational signal predominance; inability to distinguish early vs. late sepsis phases
ARDS	Higher oxygenation targets during mechanical ventilation	Facilitation of oxygenation in early shunt-dominated physiology	Potential amplification of oxygen-induced lung injury in later stages	Pooling of early and late ARDS; variable FiO_2_ titration protocols
Neurocritical care (e.g., traumatic brain injury)	Liberal oxygen strategies	Prevention of cerebral hypoxia during acute injury	Cerebral vasoconstriction and reduced cerebral blood flow with hyperoxia	Limited randomized data; short exposure assessment; lack of neurological outcome granularity
COVID-19 related respiratory failure	Liberal oxygen therapy	Initial stabilization of hypoxemic respiratory failure	Possible differential harm depending on compliance and perfusion phenotypes	Heterogeneous disease phenotypes; evolving standards of care; exposure overlap

ARDS: Acute Respiratory Distress Syndrome; COVID-19: Coronavirus Disease 2019; FiO_2_: Fraction of Inspired Oxygen; ICU: Intensive Care Unit; PaO_2_: Partial Pressure of Arterial Oxygen; SpO_2_: Peripheral Capillary Oxygen Saturation.

**Table 2:** Phenotype-oriented considerations for precision oxygen therapy in critically ill patients *.

Clinical Phenotype	Dominant Physiological Concern	Oxygen-Related Risk Profile	Conceptual Oxygenation Considerations	Nature of Recommendation
Chronic hypercapnia/COPD	Ventilation–perfusion mismatch; CO_2_ retention	High susceptibility to oxygen-induced hypercapnia and acidosis	Avoid unnecessarily high PaO_2_; prioritize avoidance of hyperoxia and excessive FiO_2_	Hypothesis-generating, physiology-based
Traumatic brain injury/acute neurological injury	Cerebral blood flow regulation	Hyperoxia-induced cerebral vasoconstriction	Avoid sustained PaO_2_ levels associated with reduced cerebral perfusion; minimize hyperoxic exposure	Hypothesis-generating, physiology-based
Sepsis and septic shock	Microvascular and mitochondrial dysfunction	Oxidative stress outweighing oxygen delivery benefits in some phases	Individualize oxygen targets according to perfusion status and disease phase	Hypothesis-generating, context-dependent
Early ARDS	Severe shunt physiology	Risk of hypoxemia predominates early	Higher oxygen requirements may be unavoidable early; avoid prolonged extreme hyperoxia	Hypothesis-generating, stage-specific
Late ARDS	Reduced recruitability; inflammation	Increased vulnerability to oxygen toxicity	Lower oxygen targets may be tolerated; prioritize minimizing cumulative hyperoxic exposure	Hypothesis-generating, stage-specific
COVID-19 respiratory phenotypes	Variable compliance and perfusion patterns	Differential susceptibility to hyperoxia	Oxygen targets should reflect underlying phenotype rather than uniform thresholds	Hypothesis-generating, phenotype-dependent
Postoperative/low-risk ICU patients	Preserved oxygen delivery	Lower immediate risk of hypoxemia	Avoid routine liberal oxygen administration without indication	Hypothesis-generating, precautionary

* These considerations are hypothesis-generating and non-prescriptive and are not intended as clinical recommendations or guideline directives. ARDS: Acute Respiratory Distress Syndrome; CO_2_: Carbon Dioxide; COPD: Chronic Obstructive Pulmonary Disease; COVID-19: Coronavirus Disease 2019; FiO_2_: Fraction of Inspired Oxygen; ICU: Intensive Care Unit; PaO_2_: Partial Pressure of Arterial Oxygen.

## Data Availability

No new data were created or analyzed in this study. Data sharing is not applicable to this article.
